# Assessment of genetic diversity, population structure and wolf-dog hybridisation in the Eastern Romanian Carpathian wolf population

**DOI:** 10.1038/s41598-023-48741-x

**Published:** 2023-12-19

**Authors:** Anne Jarausch, Alina von Thaden, Teodora Sin, Andrea Corradini, Mihai I. Pop, Silviu Chiriac, Andrea Gazzola, Carsten Nowak

**Affiliations:** 1 Conservation Genetics Group, Senckenberg Research Institute and Natural History Museum Frankfurt, Clamecystraße 12, 63571 Gelnhausen, Germany; 2 Department of Biological Sciences, Johann Wolfgang Goethe-University, Biologicum, Max-von-Laue-Straße 9, 60438 Frankfurt am Main, Germany; 3 LOEWE Centre for Translational Biodiversity Genomics (LOEWE-TBG), Senckenberganlage 25, 60325 Frankfurt am Main, Germany; 4 Department of Systems Ecology and Sustainability, Faculty of Biology, University of Bucharest, Splaiul Independentei 91-95, 050095 Bucharest, Romania; 5 Association for the Conservation of Biological Diversity, Ion Creanga 12, 620083 Focsani, Romania; 6 Environmental Protection Agency, Vrancea County, Dinicu Golescu 2, 620106 Focsani, Romania; 7 Present Address: Animal Ecology Unit, Research and Innovation Centre, Fondazione Edmund Mach, Via Edmund Mach 1, 38098 San Michele all’Adige, TN Italy; 8 Present Address: NBFC, National Biodiversity Future Center, 90133 Palermo, PA Italy

**Keywords:** Ecology, Genetics, Molecular biology, Zoology

## Abstract

The Carpathian Mountains have been constantly inhabited by grey wolves and present one of the largest distribution areas in Europe, comprising between 2300 and 2700 individuals in Romania. To date, however, relatively little is known about the Romanian wolf population. We aimed to provide a first assessment of genetic diversity, population structure and wolf-dog hybridisation based on 444 mostly non-invasively collected samples in the Eastern Romanian Carpathians. Pack reconstruction and analysis of population genetic parameters were performed with mitochondrial DNA control-region sequencing and microsatellite genotyping. We found relatively high levels of genetic diversity, which is similar to values found in previous studies on Carpathian wolves from Poland and Slovakia, as well as to the long-lasting Dinaric-Balkan wolf population. We found no significant population structure in our study region, suggesting effective dispersal and admixture. Analysis of wolf-dog hybridisation using a Single Nucleotide Polymorphism panel optimised for hybrid detection revealed low rates of admixture between wolves and domestic dogs. Our results provide evidence for the existence of a genetically viable wolf population in the Romanian Carpathians. The genetic data obtained in this study may serve as valuable baseline information for the elaboration of monitoring standards and management plans for wolves in Romania.

## Introduction

Conservation genetics emerged since the 1970s as a tool to help preserving genetic diversity in management and decision-making of wild populations^1^. As it is often difficult or impossible to obtain large numbers of high-quality genetic samples, non-invasive sampling of various DNA sources including scat, urine, hairs or saliva, is increasingly used to study population parameters of rare or elusive species (reviewed in^2^). Non-invasive genetics now constitutes a key part in many monitoring and management activities for assessing the status of wild populations. For instance, extensive genetic surveys have been implemented to study genetic diversity in rare and elusive large carnivore populations worldwide, such as the Iberian lynx *Lynx pardinus*^3^, the Mexican grey wolf *Canis lupus baileyi*^4^ or the Apennine brown bear *Ursus arctos marsicanus*^5^. Although large carnivores are often regarded as threats to human safety, livestock and wild game species, they increasingly become recognised as apex predators that play key roles in various ecosystems^6^.

The grey wolf *Canis lupus* Linnaeus, 1758 was eradicated from most parts of Europe showing the largest reduction in population size and range in the 1950–1970s^7^. Due to protective legislation and socioeconomic changes, wolves are naturally recolonising parts of their former European range within several human-dominated cultural landscapes of Western and Central Europe^8^.

Genetic diversity is considered to be essential for the long-term adaptive potential of wolf populations, especially the ability to respond to changing environmental conditions, disease resistance and anthropogenic influences, such as climate or habitat change, fluctuations in prey availability or occurring communicable diseases^9^. Erosion of genetic diversity and inbreeding can lead to an increased extinction risk of wildlife populations, underlining the importance of assessing genetic diversity parameters for conservation efforts^10^. While newly establishing small wolf populations may face genetic and demographic bottlenecks along with other challenges such as inbreeding and loss of genetic diversity^11^, gene flow from the source populations and adjacent populations supports the long-term viability of these expanding populations.

The evolutionary history of wolf-like canids is characterised by interspecific gene flow across the genus *Canis*^12^. Currently, the hybridisation between wolves and domestic dogs is considered as a serious conservation threat, as it may erode the genetic integrity and adaption of European wolf populations in the long term^9,13^. Regional wolf populations in Southern and Eastern Europe are particularly affected, where feral and free-ranging dogs are common^14^. There is thus an urgent need to implement appropriate hybrid management with integration of scientific, legal, social, ethical and political perspectives to reduce the spread of free-ranging dogs and wolf-dog hybrids^15^.

Romania is a member state in the European Union (EU) since 2007. Hence, the wolf is listed in Annex II and IV under the conservation legislation of the EU Habitats Directive (Council Directive 92/43/EEC), with the overall goal to maintain the ‘Favourable Conservation Status, FCS’ (Article 2). Romanian legislation included exemptions under Article 16 allowing for a restricted cull by hunters, which were integrated in the decades-old wildlife management system. However, hunting of wolves was eventually banned by the Romanian government in October 2016^16^, case by case derogations being approved for damage prevention. As EU member state, Romania has to continuously monitor the conservation status of the wolf, which is still based on the interpretation of surveys carried out in the distinct wildlife management units through snow tracking in winter^17–19^. The drawback of this approach is that wolves are not individually identifiable, which increases the risk of double counts. Thus, some studies have queried the robustness of such wolf population size estimates^16,18,19^.

While the apex predator has been historically eradicated from vast areas, the Carpathian Mountains were always inhabited by wolves and present one of the largest distributions in Europe, comprising between 2300 and 2700 individuals in Romania (19.2–22.5% of wolves in Europe)^7^. Despite the fact that the Carpathian wolf population has formed an important stronghold for species persistence in Europe, relatively little is known about the Carpathian wolves in Romania, including sound estimates of population census^17,18^, pack structure, potential hybridisation with dogs or genetic differentiation from other European populations. Previous genetic studies mostly included samples from the North-Western part of the Carpathian wolf population in Poland, Slovakia or Ukraine^20–26^. The gap in profound scientific knowledge of the Eastern Carpathian wolf population is even more striking, as this region is considered to be important for the long-term viability of wolves due to the large unfragmented geographical area, and furthermore providing a corridor between populations in the North and the South^9^.

To analyse genetic diversity, population structure and hybridisation in this important but understudied part of the European wolf distribution range, we conducted a first genetic assessment of wolves within the Central Eastern Romanian Carpathian Mountains, focusing on four core study areas and the surrounding area (PVSO [Putna-Vrancea Natural Park, Soveja SCI, Oituz SCI], HHM [Herculian SCI, Harghita-Madaras SCI], MCG National Park and VNT [Vanatori-Neamt Natural Park] were chosen as study areas for a field study in the WolfLife project LIFE13NAT/RO/000205 aiming at testing various methods for wolf monitoring in the Eastern Carpathian Mountains). Specifically, we investigated if wolves in the Eastern Romanian Carpathians showed (1) similar levels of genetic diversity as previous studies found for wolves in other regions of the Carpathian Mountains, (2) population structure, providing evidence for dispersal barriers, and (3) whether the known occurrence of free ranging dogs, such as stray dogs or livestock guardian dogs may have led to elevated hybridisation rates.

To answer these questions, we applied a combination of mitochondrial DNA sequencing as well as microsatellite- and Single Nucleotide Polymorphism-based genotyping methods based on mostly non-invasively collected wolf samples.

## Results

### Genetic diversity

**Figure 1:**
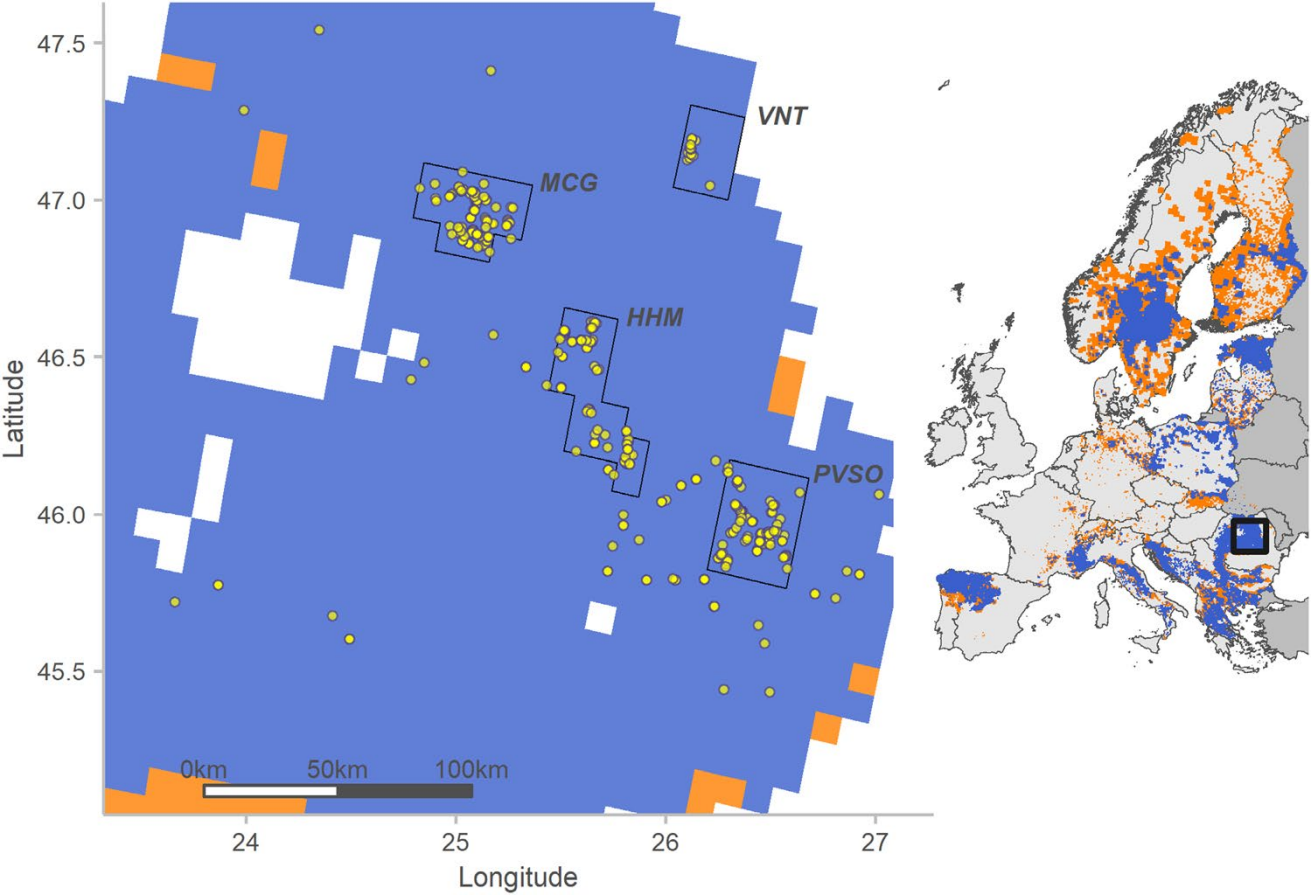
Genetically identified wolf samples (*n* = 378; yellow circles) collected in the four core study areas PVSO, HHM, MCG, VNT (black lines) and in the surrounding area. The smaller map (right) shows the study area (solid black frame) in the Eastern Romanian Carpathians. Wolf distribution across Europe in the period 2012–2016 with permanent occurrence (blue) and sporadic occurrence (orange) is shown according to^27^.

We analysed 444 mostly non-invasive samples collected between 2011 and 2017 across the Eastern Romanian Carpathians within the study region (four core study areas and the surrounding area, Fig. 1). Individual genotype assignments based on 13 microsatellite loci were obtained for 296 samples of 126 wolf individuals (50 females, 75 males, RW016 unidentified sex).

Each wolf individual was genotyped 2.35 times on average (range = 1–17 genotypes per individual, 64 of 126 individuals genotyped once). Samples showed high average PCR amplification success (91.1% rate). Average rates of allelic dropout and false alleles were low (5.7% and 0.8%, respectively). No evidence of frequency distortion through large allele drop-outs or stutter peaks was identified for the 13 microsatellite loci. Potential occurrence of null alleles was found for the loci FH2017, FH2096 and vWF. The probability of identity (PID) was 1.2 × 10^−13^ and probability of identity between siblings (PIDsib) was 1.2 × 10^−05^ for all identified wolf individuals, indicating that the set of microsatellite loci was sufficient for individualisation even among siblings. PID and PIDsib < 0.0001 was reached with at least five and 11 markers, respectively.

Between three and 15 alleles were genotyped among the 13 loci with a mean of 7.31 alleles per locus across all wolf individuals (*n* = 126). Mean observed heterozygosity (*Ho*) was 0.69 ± 0.04 and the mean unbiased expected heterozygosity (*uHe*) was 0.73 ± 0.04. When testing all genotyped wolves, three of the 13 loci (vWF, FH2137 and FH2161) deviated significantly from HWE (*p* < 0.05).

Mitochondrial DNA haplotype diversity was assessed by sequencing short fragments of the mtDNA control-region by using two different sets of primers resulting in stretches with a length of 250 or 390 bp. Species determination was successful for a total of 406 out of 444 (91.4%) samples, with 378 samples from wolves and 32 samples from dogs and seven other mammal species (see Supplementary Results and Tables S1–S5 for details).

**Figure 2:**
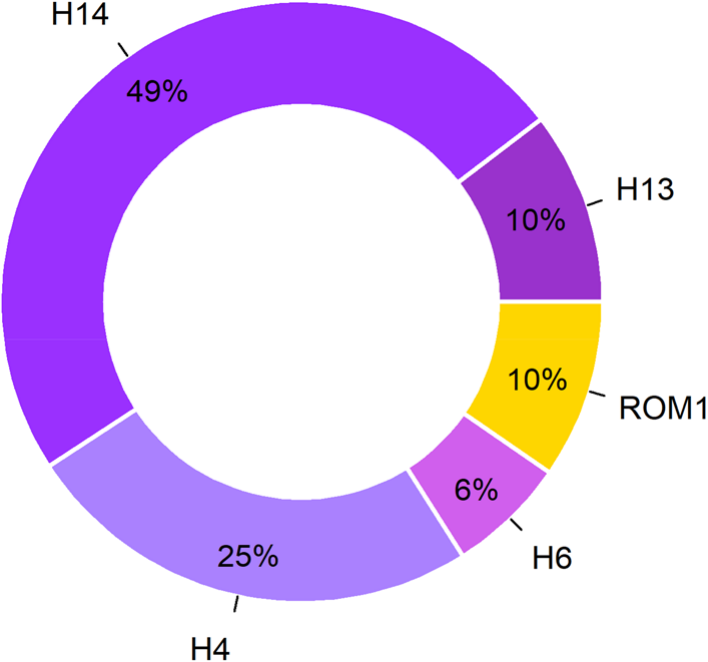
Frequencies of the five identified mtDNA control-region haplotypes of all 125 wolf individuals in the Eastern Romanian Carpathians.

Five mtDNA wolf haplotypes were identified in 125 wolf individuals (no haplotype could be identified for the wolf RW052m): H14 (most frequent, 49%), followed by H4 (25%) and H13 (10%) according to^28^ as well as one previously undescribed haplotype in Romania (here typed as ROM1, 10%) (Fig. 2). Interestingly, haplotype ROM1 matched with sequences (361 bp; ID: MH891616^29^ and 356 bp; ID: MK129178 & MK129179^30^) obtained from museum specimens referable to the extinct wolf population of Sicily and with a sequence obtained from an individual discovered in Southern France in 1954 CE (361 bp; ID: OM743388^31^). Another haplotype, H6^28^, was found in eight wolf samples (6%) collected in the surrounding area but not within the four core study areas.

### Relatedness and population structure

**Figure 3:**
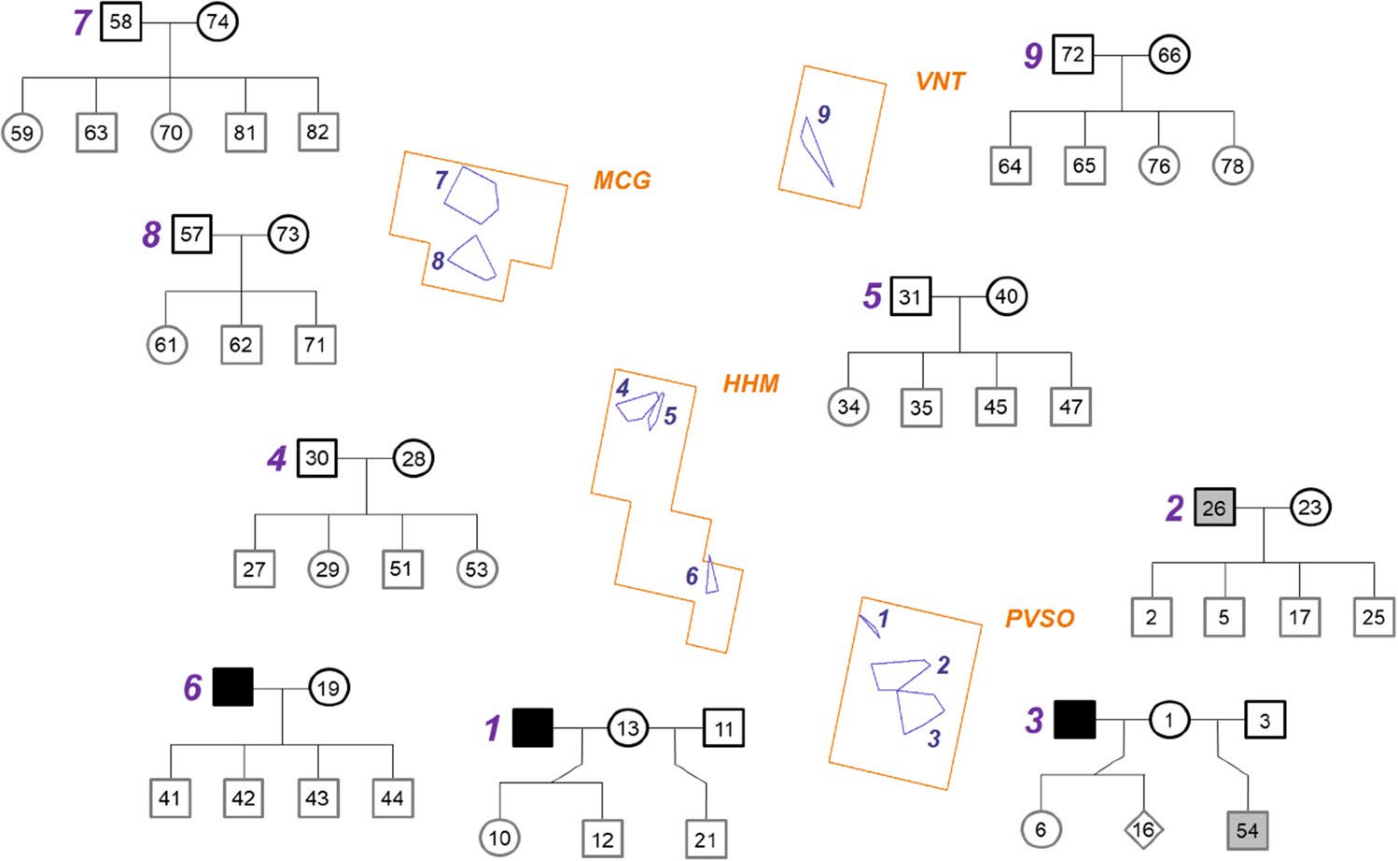
Spatial distribution of packs (purple polygons labelled with numbers) in the four core study areas PVSO, HHM, MCG and VNT (orange lines). Minimum convex polygons include all samples of individuals of each identified pack. Pedigrees of the inferred packs (labelled with purple numbers of the respective packs) consisting of the breeding individuals (black outlines) and their pups (grey outlines). Males are represented by squares, females by circles and the individual for which sexing was not successful by a diamond. Non-sampled males are filled with black and males which were collected in the following winter are filled with grey.

Genetic parentage analysis in COLONY resolved eleven parent–offspring relationships consisting of one or both parents and between one to five pups within the four core study areas (Fig. 3, packs 1–9). COLONY results were cross-checked by manual comparison of genotypes in combination with the number of detections of each individual, as breeding animals of a pack are scent-marking their home range (territory) and are usually identified more often than their offspring. Based on the spatial clustering of individuals, the different wolf territories were identified and separated from adjacent wolf packs. Pack reconstruction and spatial mapping of samples revealed nine territories, inhabited by wolf packs (Fig. 3). In two territories, a male and a female were each identified repeatedly (RW008m and RW004f; RW060m and RW080f), suggesting that these individuals likely inhabited these areas as wolf pairs without offspring. The pedigree analysis revealed that the breeding males had changed in pack 1 and 3 over time, while the breeding females with their offspring of the previous mating still inhabited the territories. We further detected 23 individuals in VNT, HHM and MCG, which appeared to be unrelated or were sampled along the borders among territories or in areas more distant to any inferred pack or pair. These individuals were considered as floaters or members of other packs or pairs.

A reduced dataset of unrelated wolf individuals for population structure analyses contained 47 individuals, including both wolves from the core study areas and wolves collected in the surrounding area (see supporting datasets for details). Wolf genotypes of this subset were in Hardy–Weinberg equilibrium (HWE; *p* > 0.05). STRUCTURE and GENELAND analyses as well as the Bayesian information criterion revealed no population structure (Fig. S1).

### Wolf-dog hybridisation

DNA from 127 putative wolf individuals (52 tissue and 75 non-invasive samples) and ten reference dogs (4 tissue and six non-invasive samples) identified by the preceding microsatellite analysis were genotyped by using a panel of 96 ancestry informative Single Nucleotide Polymorphism (SNP) markers to clearly differentiate between wolves, dogs and potential wolf-dog hybrids. Three individuals (RW025m, RW042m and RW043m) were not included for SNP analysis due to insufficient SNP genotype quality (genotypes with < 0.60 call rates). Two of the 96 SNPs (BICF2P995528 and BICF2P1334457) were filtered out from the final dataset due to low genotyping rate (< 0.60). The average genotyping success rate (proportion of successfully genotyped loci over the 94 SNP loci for the analysed samples) was 0.97. The consistency of the genotypes was also high. When comparing results from a subset 14 replicated samples (10% of all individuals), missing data was obtained for 1–17 loci per sample (2.51% rate) and missing alleles in the replicates of one dog sample (1.44% rate).

**Figure 4:**
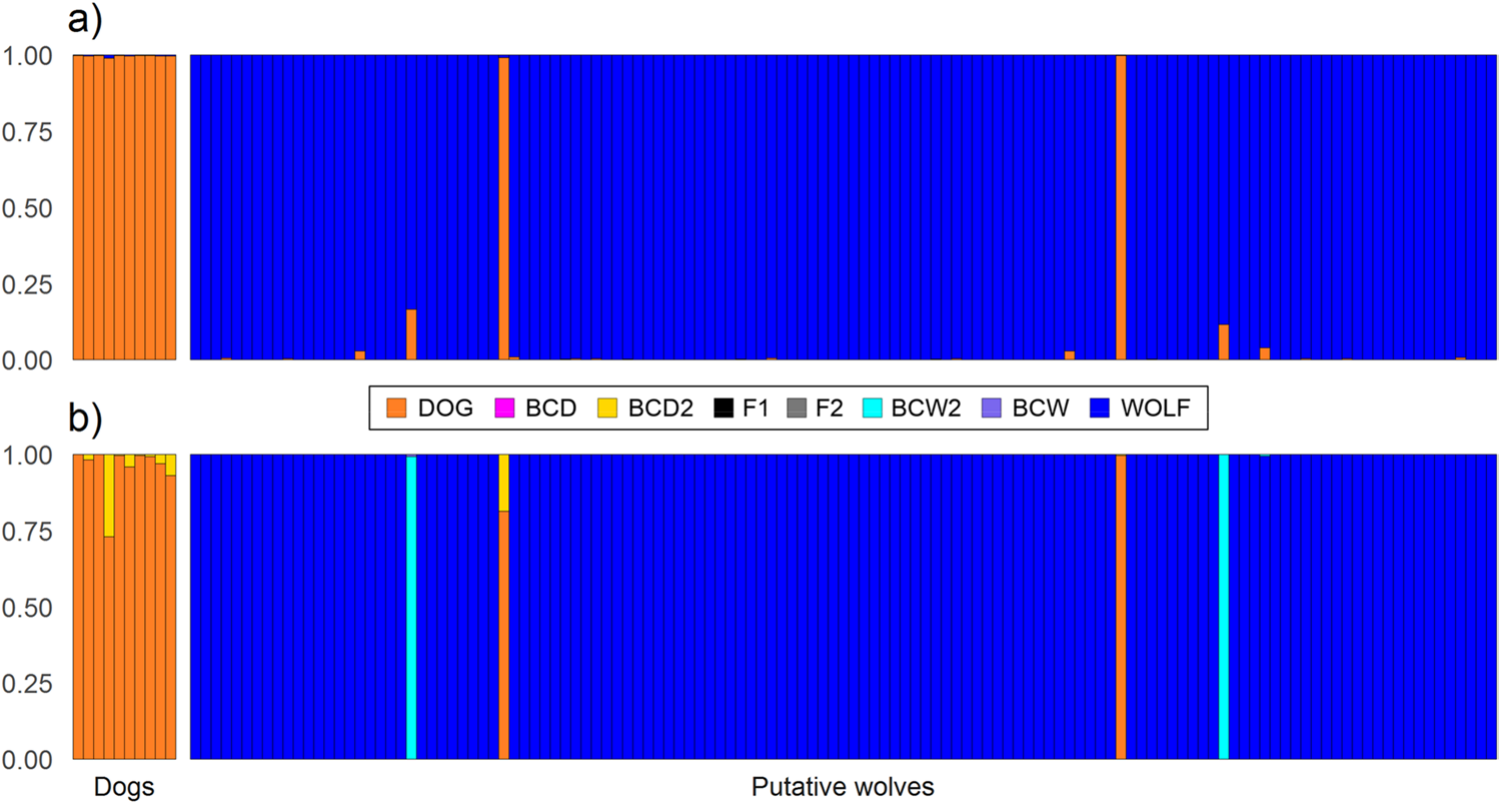
Discrimination between dogs (*n* = 10), putative wolves (*n* = 127) and detection of possible hybrids based on SNP data using a) STRUCTURE for *K* = 2 (DOG and WOLF) and b) NewHybrids with eight genealogical categories (WOLF, DOG, F1, F2, first generation backcross to wolf (BCW), second generation backcross to wolf (BCW2), first generation backcross to dog (BCD) and second generation backcross to dog (BCD2)). Each individual is represented by a vertical bar divided into coloured segments according to a) the membership proportions of the two inferred clusters and b) to its parental or hybrid category. The hybrid categories F1, F2, BCW and BCD are not represented by coloured segments in the bars as individuals showed assignment probabilities < 0.008.

Bayesian STRUCTURE analysis of the 127 putative wolves and the ten reference dogs indicated that the most likely number of genetic clusters was *K* = 2 (Fig. 4a). All reference dogs were assigned to one cluster with an average probability of 0.997 ranging between 0.991 and 0.999. Among the 127 putative wolf genotypes detected in the study region, 120 individuals were clearly assigned to the wolf cluster and two to the dog cluster, all with probabilities > 0.99, respectively. Three individuals showed slightly lower but still significant assignment probabilities to the wolf cluster from 0.96 to 0.971, in accordance with previous knowledge^32^. Two individuals had probabilities of 0.834 and 0.884, indicating signs of admixture.

Differentiation between wolves and dogs in the study region was confirmed by a Principal Coordinate Analysis (PCoA), which separated the putative wolves and reference dogs into two clusters along the first PC1-axis (59.35% of the total variation, Suppl. Fig. S2). The two individuals inferred as dogs in the STRUCTURE analysis clustered together with the reference dog samples, while the two individuals showing signs of admixture were placed close to the wolf cluster. Results of the PCoA and STRUCTURE analysis were supported by Bayesian assignment of individual genotypes using NewHybrids (Fig. 4b). Most putative wolves were assigned to the wolf category with a probability > 0.995, two individuals assigned to the dog category with probabilities of 0.814–0.997, while two individuals (RO022m in a tissue sample and RW056m in a scat sample) had the highest probabilities to be second generation backcrosses to wolf (BCW2; 0.992–1.0).

## Discussion

This study is the first genetic assessment of wolves within the Eastern Romanian Carpathian Mountains using mtDNA haplotype sequencing, microsatellite genotyping and SNP-based wolf-dog hybrid identification. Microsatellite diversity measures in the Romanian wolves (*Ho* = 0.69) are comparable to other studies on Carpathian wolves in Poland and Slovakia (*Ho* = 0.69,^23^; *Ho* = 0.67,^25^) and similar to the long-lasting Dinaric-Balkan wolf population (*Ho* = 0.73,^33^). Our findings fit the large-scale spatial trends of genetic diversity across Europe^9^ with the lowest levels of genetic diversity found in the somewhat isolated Southwestern wolf populations (Italian/Alpine population, *Ho* = 0.57,^34^ and the Iberian population, *Ho* = 0.52,^35^) and increasing genetic diversity towards the Northeast (Estonia and Latvia, *Ho* = 0.75,^36^; European Russia, *Ho* = 0.74,^37^).

Microsatellite genotypes of all wolves deviated significantly from HWE and *Ho* was lower compared to *uHe*, likely caused by the composition of the sample set including closely related individuals of the different wolf packs.

Four of the identified mtDNA haplotypes have either been already described for Romania (H14 and H4) or are at least known to occur widespread across Eastern and South-Eastern Europe (H14, H13, H6 and H4)^26,28^. Somewhat more surprising was the fact that haplotype ROM1 matched with sequences obtained from museum specimens referable to the extinct wolf population of Sicily^29,30^. However,^30^ and^29^ showed that this wolf haplotype belongs to haplogroup 2 described in^28^. The 361 bp fragment of haplotype ROM1 fully matched to a Sicilian haplotype (ID: MH891616^29^) and a French haplotype (ID: OM743388^31^) but differed by only one substitution from a French (ID: OM743385^31^), a Bulgarian (ID: KU696388), a Polish (ID: KF661045) and the wolf haplotype H6 (this study and^28^). This finding documents the low grade of previous genetic knowledge on wolves in Romania. Moreover, our results indicate that haplotype ROM1 was shared between different regions across Europe before human-induced local extinctions and the strong loss of genetic diversity in Western Europe occurred during the last 200 years, which has already been suggested for most other European wolf haplotypes^31,38^. Future studies covering longer mtDNA sequences or ideally complete mitogenomes from all haplogroup 2 wolf populations may help to better understand the evolutionary dynamics and phylogeographic structure of European wolves.

We found clear patterns of social structure across the study area, as kinship analyses suggested the presence of nine wolf packs. A wolf pack is usually organised as a family unit comprising a breeding pair and their offspring of the present and the previous year^39^. Several studies found that breeding pairs may hold their territory and reproduce for several years. However, breeding pair bonds persist on average only about two years, e.g., in the recolonising wolf populations in Scandinavia^40^ and Germany^41^ or in the heavily exploited Białowieża population on the Polish-Belarussian border^42^. It is also known that mostly males immigrate into pre-existing packs, becoming the new breeding male, while female offspring often fill territories close to their natal pack or the territory is taken over by female offspring of the parental breeding pair (e.g.,^41–44^). As dissolution of wolf pairs has been found after the death of one or both breeders^40^, we assume that the reproductions with new breeding males in PVSO (pack 1 and 3) were not resulting from active mate choice by the female but rather a reaction to losing her partner.

Results of population structure analyses based on unrelated wolves (*n* = 47) were consistent and revealed no genetic structure, which is in line with a previous genetic study on wolves in Romania^45^, suggesting high dispersal and diversity due to the existence of vast natural habitats with few anthropogenic disturbances, such as settlements or traffic infrastructure. One needs to consider, however, that our study was done on a local scale, and hence we likely missed potential landscape barriers or filters on larger spatial scales, e.g., resulting from isolation-by-distance.

Hybridisation of wolves and domestic dogs is considered a serious threat to long-term genetic integrity in several European wolf populations^9^. A previous population size estimate^18^ suggests that the Romanian wolf population may suffer high losses through anthropogenic mortality (hunting, poaching, roadkill and diseases transmitted by dogs). These fatal losses disturb the intact social structure of wolf packs, forcing them to accept individuals from outside of the pack^42^, which may elevate hybridisation risk.

We used 94 ancestry informative SNPs for the identification of possible hybrids to achieve a high resolution in wolf-dog-hybrid discrimination. In this study, all individuals (except of RW025m, RW042m and RW043m, see “Results”) were identified as wolves, except of two individuals identified as dogs and only two individuals identified as later-generation hybrids (second generation backcross to wolf). In the study of^32^, a selected number of individuals consisting of samples from wolves, dogs and a hybrid were analysed including a larger sample set of wolves sampled across Europe as well as dogs and suspected wolf-dog hybrids. In this more comprehensive study, samples were assigned to the same parental/hybrid category, which supports the non-hybrid status of individuals considered as wolves or dogs and the hybrid status of the two suspected individuals.

The presence of feral and free-ranging dogs (stray or livestock guardian dogs) in wolf habitats may increase the chance of wolf-dog hybridisation events. Notably, wolf diet and prey selection analyses carried out in the study area PVSO revealed that dogs present an important food source for wolves especially during summer when the dogs are present on high altitude pastures to defend the herds of sheep (mostly one or two livestock guardian dogs and several small-sized mixed-breed dogs per herd)^46^. The fact that dogs stay in or near settlements during winter, makes it more difficult for wolves to reach dogs as food source but also as potential breeding partners, which may explain the observed low hybridisation rate despite of the frequent occurrence of free-ranging dogs in the study region. More samples from a wider area need to be collected across the wolf distribution range to obtain reliable estimates of wolf-dog hybridisation rates in Romania.

While the Carpathian wolf population displays an important stronghold for the species’ long-term persistence in Europe^9^, relevant data on the genetic diversity and structure of Carpathian wolves in Romania is still scarce. Our first regional genetic assessment of wolves within the Eastern Romanian Carpathian Mountains shows relatively high levels of genetic diversity due to social pack structure and high dispersal of wolves within the study region. We found no evidence for genetic risks, e.g., through elevated dog introgression. In conclusion, our study supports the importance of the Romanian wolf population for wolf conservation in Europe.

Since the minimum number of wolves and the number of packs can be used for further population estimates^47^, the data from our study might be useful to generate reliable wolf population size estimates in Romania. We hope that wolf management in Romania may benefit from our findings, helping to make well-informed decisions by integrating comprehensive genetic analyses based on non-invasive genetic sampling when aiming for future estimation of population size and genetic status. Further studies on wolves in the Carpathian Mountains are needed to obtain more robust information required for sound conservation planning, especially on large-scale population structure, temporal and spatial distribution of genetic diversity and wolf-dog hybridisation rates.

## Methods

### Study area and sample collection

The Eastern Romanian Carpathian Mountains were not reliably monitored and sound estimates of wolf numbers or pack distributions were missing for this area. Therefore, a spatially balanced sampling design was implemented to ensure the detection and collection of a representative number of samples. Following the EU recommendations to use a spatially standardised data collection method across Europe^48^, six to twelve equally sized sampling units were selected by placing the 10 × 10 km EEA grid over a topographical map. The genetic study was carried out in the Eastern Romanian Carpathian Mountains including four core study areas with a total surface of 4000 km^2^: (1) PVSO [Putna-Vrancea Natural Park, Soveja SCI, Oituz SCI], 1200 km^2^ with 12 sampling units; (2) HHM [Herculian SCI, Harghita-Madaras SCI], 1200 km^2^ with 12 sampling units; (3) MCG [Muntii Calimani-Gurghiu SCI], 1000 km^2^ with 10 sampling units; and (4) VNT [Vanatori-Neamt Natural Park], 600 km^2^ with 6 sampling units (Fig. 1).

The core study areas were surveyed exclusively during the winter season (i.e., from November to April) in subsequent years: PVSO 2014–2015, HHM 2015–2016, MCG and VNT 2016–2017. The winter was chosen as the social structure of wolf packs and thus the wolf population is relatively stable at this period of the year (pup mortality is high during the first months of life and pups have gained in body size and are moving together with the rest of the natal pack). Moreover, the wintry conditions are best suited for detecting wolf presence and assessing wolf territories (e.g., identifying scent-marks, snow-tracking, collecting DNA samples). A consistent and spatially standardised sampling effort was ensured, by setting a minimum cumulative transect length of 20 km within each sampling unit (10 × 10 km grid cell). Transects were selected along the existent forest roads and footpaths and were surveyed on foot. Each transect was covered at least three times per survey season by splitting the six-month survey seasons into three two-month survey seasons (November–December, January–February, and March–April, respectively). In total, 444 mostly non-invasive wolf samples were collected (226 scats, 102 urine samples, 39 hairs, and one saliva sample from a kill). Additional samples were opportunistically collected in the surrounding area between 2011 and 2017, including 66 tissue samples obtained from legally harvested wolves and one roadkill. Ten samples from domestic dogs (three saliva, three scat and four tissue samples) were collected between January 2016 and March 2017 to detect putative wolf-dog hybrids in the dataset.

Urine samples were stored in 33 ml of 96% ethanol by adding 15 ml of urine-snow mixture. Scat and tissue samples were stored in 96% ethanol. Hairs were collected in filter paper and then stored dry in a plastic bag. Saliva swabs were stored in 2 ml Eppendorf tubes containing 96% ethanol or collected in filter paper and then stored dry in a plastic bag. All samples were stored under dry conditions at room temperature without direct sunlight until DNA extraction.

No animals were handled or killed for this study. Non-invasive samples were collected in compliance with the respective local and national laws. No ethics approval was necessary to work with non-invasive samples or tissues from dead animals.

### Genetic analyses

The protocols and laboratory procedures for DNA extraction, mtDNA sequencing, microsatellite genotyping and sex identification have been described in^41^ and^49^. Briefly, DNA was extracted according to the different sample types using the DNeasy® Blood & Tissue Kit (Qiagen), the QIAamp DNA Stool Mini Kit (Qiagen) or the QIAamp DNA Investigator Kit (Qiagen). MtDNA haplotypes were determined through sequencing 250 or 390 base pairs (bp) of the mtDNA control-region by using two different sets of primers, WDloopL and WDloopH254^50^ or L15995^51^ and H16498^52^. Thirteen unlinked autosomal microsatellites and two sex markers were used to reconstruct the relatedness of wolf individuals according to^41^. A multiple-tube approach was applied to amplify microsatellites and sex markers including four, and for some samples, up to eight replicates per non-invasive sample to account for genotyping errors due to low quality and quantity of template DNA. DNA from individuals was subsequently genotyped using a panel of 96 ancestry informative SNP markers to clearly differentiate between wolves and dogs and for a wolf-dog hybrid assessment, which derived from the Illumina CanineHD Whole-Genome BeadChip microarray (174 K). SNP markers, protocols and laboratory procedures are described in^32^.

### Data analyses to assess genetic diversity and population structure

BLAST^53^ was used to identify possible matches between the obtained mtDNA sequences of this study and haplotypes already published in the NCBI GenBank. We followed the mtDNA wolf haplotype nomenclature of^28^ to avoid further complexity in haplotype comparison.

Matching microsatellite sample genotypes were identified using the R package DNAtools 0.1-21^54^ and the R package CONGENR^55^ was used to estimate genotyping error rates in the R programming language^56^. MICRO-CHECKER 2.2.3^57^ was used to test for scoring errors caused by stutter peaks, large allelic dropout and the presence of null alleles. Descriptive statistics of microsatellite loci such as the mean number of different alleles per locus, observed heterozygosity (*Ho*) and unbiased expected heterozygosity (*uHe*) as well as the probability of identity (PID) and probability of identity between siblings (PIDsib)^58^ were calculated using GenAlEx 6.5^59,60^. CERVUS 3.0.7^61^ was used to generate input files for the software GENEPOP 4.7.5^62,63^ to perform Hardy–Weinberg equilibrium (HWE) testing with 5000 dememorisations, 500 batches and 5000 iterations per batch. For robust pack reconstructions, we combined the genetic data (i.e., mtDNA haplotypes, microsatellite genotypes and sex) with the spatio-temporal information from the field data (i.e., collection dates, sample types and the collection localities). The pack reconstructions were supported by parentage assignments using full-pedigree likelihood methods implemented in COLONY 2.0.6.4^64^. We ran COLONY using the full likelihood method with ‘medium length of run’ and ‘medium likelihood precision’ options including all individuals. The inbreeding model was selected and mating systems for both male and female were set to ‘polygamous’.

For population structure analyses, we excluded closely related individuals based on COLONY results and manual direct genotype comparison. We investigated population structure by using the Bayesian clustering software STRUCTURE 2.3.4^65^ with an initial burn-in of 200,000 steps and 500,000 MCMCs using the admixture model and correlated allele frequencies but without prior information. Ten independent runs for each *K* = 1–10 were performed and combined using the ‘Greedy’ algorithm as implemented in CLUMPP 1.1.2^66^. The Evanno method^67^ implemented in STRUCTURE HARVESTER 0.6.94^68^ was used to determine the most likely *K* value. We also inferred the number of populations using the R package GENELAND 4.0.8^69^ by performing ten initial runs with 100,000 iterations and thinning every 100 as well as correlated allele frequency model under the spatial prior and the non-spatial prior. Individual’s posterior probability of population membership under the spatial prior has been summarized by calculating the average from the ten independent runs. Furthermore, we identified the optimal number of clusters (*K*) based on the Bayesian information criterion (BIC;^70^) using the R package ADEGENET ver. 2.1.1^71^.

### Identification of hybrids

We tested for hybridisation between wolves and dogs using a panel of 96 ancestry informative SNPs^32^. Genotyping consistency was assessed by comparing results from 14 replicated samples (10% of all individuals). We ran the Bayesian clustering algorithm implemented in STRUCTURE 2.3.4^65^, combined the data in CLUMPP 1.1.2^66^ and used STRUCTURE HARVESTER 0.6.94^68^ with the same settings described above to infer population structure. We conducted a PCoA implemented in GenAlEx 6.5^59,60^ to infer the number of clusters of the genetically close individuals. To further evaluate individuals that showed signs of admixture in the STRUCTURE and PCoA analyses, we used the software NewHybrids 1.1^72^. The software was applied to calculate the probability of belonging to eight genealogical categories: wolf, dog, F1, F2, first generation backcross to wolf (BCW), second generation backcross to wolf (BCW2), first generation backcross to dog (BCD) and second generation backcross to dog (BCD2). We ran a burn-in of 100,000 steps, followed by 500,000 sweeps under uniform prior.

### Supplementary Information

Supplementary Tables.

Supplementary Information 2.

## Data Availability

The datasets supporting the conclusions of this article are available in the Dryad repository, https://doi.org/10.5061/dryad.gb5mkkwvm.

## References

[1] Kershaw F (2022). The Coalition for Conservation Genetics: Working across organizations to build capacity and achieve change in policy and practice. Conserv. Sci. Pract..

[2] Waits LP, Paetkau D (2005). Noninvasive genetic sampling tools for wildlife biologists: a review of applications and recommendations for accurate data collection. J. Wildl. Manage..

[3] Kleinman-Ruiz D (2019). Genetic evaluation of the Iberian lynx ex situ conservation programme. Heredity.

[4] Fitak RR, Rinkevich SE, Culver M (2018). Genome-wide analysis of SNPs is consistent with no domestic dog ancestry in the endangered Mexican Wolf (*Canis lupus baileyi*). J. Hered..

[5] Ciucci P (2015). Estimating abundance of the remnant Apennine brown bear population using multiple noninvasive genetic data sources. J. Mammal..

[6] Ripple WJ (2014). Status and ecological effects of the world’s largest carnivores. Science.

[7] Chapron G (2014). Recovery of large carnivores in Europe’s modern human-dominated landscapes. Science.

[8] Cimatti M (2021). Large carnivore expansion in Europe is associated with human population density and land cover changes. Divers. Distrib..

[9] Hindrikson M (2017). Wolf population genetics in Europe: a systematic review, meta-analysis and suggestions for conservation and management. Biol. Rev..

[10] Reed DH, Frankham R (2003). Correlation between fitness and genetic diversity. Conserv. Biol..

[11] Frankham R (2005). Genetics and extinction. Biol. Conserv..

[12] Gopalakrishnan S (2018). Interspecific gene flow shaped the evolution of the genus *Canis*. Curr. Biol..

[13] Randi E (2008). Detecting hybridization between wild species and their domesticated relatives. Mol. Ecol..

[14] Boitani, L. *et al.* Key actions for large carnivore populations in Europe. In *Institute of Applied Ecology (Rome, Italy). Report to DG Environment, European Commission, Bruxelles. Contract no. 07.0307/2013/654446/SER/B3* (2015).

[15] Stronen AV (2022). Wolf-dog admixture highlights the need for methodological standards and multidisciplinary cooperation for effective governance of wild x domestic hybrids. Biol. Conserv..

[16] Popescu V, Pop M, Chiriac S, Rozylowicz L (2019). Romanian carnivores at a crossroads. Science.

[17] Kaczensky, P. *et al.* Status, management and distribution of large carnivores – bear, lynx, wolf & wolverine – in Europe. In *IUCN/SSC Large Carnivore Initiative for Europe* (2012).

[18] Popescu V, Artelle KA, Pop MI, Manolache S, Rozylowicz L (2016). Assessing biological realism of wildlife population estimates in data-poor systems. J. Appl. Ecol..

[19] Cristescu B, Domokos C, Teichman KJ, Nielsen SE (2019). Large carnivore habitat suitability modelling for Romania and associated predictions for protected areas. PeerJ.

[20] Pilot M (2006). Ecological factors influence population genetic structure of European grey wolves. Mol. Ecol..

[21] Czarnomska SD (2013). Concordant mitochondrial and microsatellite DNA structuring between Polish lowland and Carpathian Mountain wolves. Conserv. Genet..

[22] Stronen AV (2013). North-South differentiation and a region of high diversity in European wolves (*Canis lupus*). PLoS ONE.

[23] Hulva P (2018). Wolves at the crossroad: Fission-fusion range biogeography in the Western Carpathians and Central Europe. Divers. Distrib..

[24] Pilot M (2018). Widespread, long-term admixture between grey wolves and domestic dogs across Eurasia and its implications for the conservation status of hybrids. Evol. Appl..

[25] Szewczyk M (2019). Dynamic range expansion leads to establishment of a new, genetically distinct wolf population in Central Europe. Sci. Rep..

[26] Šnjegota D (2023). The role of the Caucasus, Carpathian, and Dinaric-Balkan regions in preserving wolf genetic diversity. Mamm. Biol..

[27] Kaczensky, P. *et al.* Distribution of large carnivores in Europe 2012–2016: Distribution maps for Brown bear, Eurasian lynx, Grey wolf, and Wolverine. *Dryad. *https://datadryad.org/stash/dataset/doi:10.5061/dryad.pc866t1p3 (2021).

[28] Pilot M (2010). Phylogeographic history of grey wolves in Europe. BMC Evol. Biol..

[29] Reale S (2019). Biodiversity lost: The phylogenetic relationships of a complete mitochondrial DNA genome sequenced from the extinct wolf population of Sicily. Mamm. Biol..

[30] Angelici FM (2019). The Sicilian wolf: genetic identity of a recently extinct insular population. Zool. Sci..

[31] Doan K (2023). Evolutionary history of the extinct wolf population from France in the context of global phylogeographic changes throughout the Holocene. Mol. Ecol..

[32] Harmoinen J (2021). Reliable wolf-dog hybrid detection in Europe using a reduced SNP panel developed for non-invasively collected samples. BMC Genom..

[33] Šnjegota D (2021). Population genetic structure of wolves in the northwestern Dinaric-Balkan region. Ecol. Evol..

[34] Fabbri E (2014). Genetic structure of expanding wolf (*Canis lupus*) populations in Italy and Croatia, and the early steps of the recolonization of the Eastern Alps. Mamm. Biol..

[35] Sastre N (2011). Signatures of demographic bottlenecks in European wolf populations. Conserv. Genet..

[36] Hindrikson M (2013). Spatial genetic analyses reveal cryptic population structure and migration patterns in a continuously harvested grey wolf (*Canis lupus*) population in North-Eastern Europe. PLoS ONE.

[37] Korablev MP, Korablev NP, Korablev PN (2021). Genetic diversity and population structure of the grey wolf (*Canis lupus* Linnaeus, 1758) and evidence of wolf × dog hybridisation in the centre of European Russia. Mamm. Biol..

[38] Dufresnes C (2018). Howling from the past: Historical phylogeography and diversity losses in European grey wolves. Proc. R. Soc. B: Biol. Sci..

[39] Mech, L. D. & Boitani, L. Wolf social ecology. In *Wolves: behavior, ecology, and conservation* (ed. Mech, L. D. & Boitani, L.) 1–34 (University of Chicago Press, 2003).

[40] Milleret C (2017). Let’s stay together? Intrinsic and extrinsic factors involved in pair bond dissolution in a recolonizing wolf population. J. Anim. Ecol..

[41] Jarausch A, Harms V, Kluth G, Reinhardt I, Nowak C (2021). How the west was won: genetic reconstruction of rapid wolf recolonization into Germany’s anthropogenic landscapes. Heredity.

[42] Jędrzejewski W (2005). Genetic diversity and relatedness within packs in an intensely hunted population of wolves *Canis lupus*. Acta Theriol..

[43] VonHoldt BM (2008). The genealogy and genetic viability of reintroduced Yellowstone grey wolves. Mol. Ecol..

[44] Caniglia R, Fabbri E, Galaverni M, Milanesi P, Randi E (2014). Noninvasive sampling and genetic variability, pack structure, and dynamics in an expanding wolf population. J. Mammal..

[45] Ericson HS (2020). Genome-wide profiles indicate wolf population connectivity within the eastern Carpathian Mountains. Genetica.

[46] Sin T, Gazzola A, Chiriac S, Rîșnoveanu G (2019). Wolf diet and prey selection in the South-Eastern Carpathian Mountains, Romania. PLoS ONE.

[47] Stenglein JL, Waits LP, Ausband DE, Zager P, Mack CM (2010). Efficient, noninvasive genetic sampling for monitoring reintroduced Wolves. J. Wildl. Manage..

[48] Annoni, A., Bernard, L., Lillethun, A., Ihde, J. & Gallego, J. Short proceedings of the 1st European workshop on reference grids. In *1st workshop on European reference grids, Ispra, Italy, 2003*. (JRC-Institute for Environment and Sustainability, 2004).

[49] Lesniak I (2017). Population expansion and individual age affect endoparasite richness and diversity in a recolonising large carnivore population. Sci. Rep..

[50] Caniglia R, Fabbri E, Mastrogiuseppe L, Randi E (2013). Who is who? Identification of livestock predators using forensic genetic approaches. Forens. Sci. Int. Genet..

[51] Taberlet P, Bouvet J (1994). Mitochondrial-DNA polymorphism, phylogeography, and conservation genetics of the brown bear ursus-arctos in Europe. Proc. R. Soc. B: Biol. Sci..

[52] Fumagalli L, Taberlet P, Favre L, Hausser J (1996). Origin and evolution of homologous repeated sequences in the mitochondrial DNA control region of shrews. Mol. Biol. Evol..

[53] Altschul SF, Gish W, Miller W, Myers EW, Lipman DJ (1990). Basic local alignment search tool. J. Mol. Biol..

[54] Tvedebrink T, Eriksen PS, Curran JM, Mogensen HS, Morling N (2012). Analysis of matches and partial-matches in a Danish STR data set. Forens. Sci. Int. Genet..

[55] Lonsinger RC, Waits LP (2015). ConGenR: rapid determination of consensus genotypes and estimates of genotyping errors from replicated genetic samples. Conserv. Genet. Resour..

[56] R Core Team. R: A language and environment for statistical computing. *R Foundation for Statistical Computing* (Vienna, Austria, 2021). https://www.R-project.org/.

[57] van Oosterhout C, Hutchinson WF, Wills DPM, Shipley P (2004). MICRO-CHECKER: software for identifying and correcting genotyping errors in microsatellite data. Mol. Ecol. Notes.

[58] Waits LP, Luikart G, Taberlet P (2001). Estimating the probability of identity among genotypes in natural populations: cautions and guidelines. Mol. Ecol..

[59] Peakall R, Smouse PE (2006). GENALEX 6: genetic analysis in Excel. Population genetic software for teaching and research. Mol. Ecol. Resour..

[60] Peakall R, Smouse PE (2012). GenAlEx 6.5: genetic analysis in Excel. Population genetic software for teaching and research—an update. Bioinformatics.

[61] Kalinowski ST, Taper ML, Marshall TC (2007). Revising how the computer program CERVUS accommodates genotyping error increases success in paternity assignment. Mol. Ecol..

[62] Raymond M, Rousset F (1995). GENEPOP (version 1.2): population genetics software for exact tests and ecumenicism. J. Hered..

[63] Rousset F (2008). Genepop’007: a complete reimplementation of the Genepop software for Windows and Linux. Mol. Ecol. Resour..

[64] Jones OR, Wang J (2010). COLONY: a program for parentage and sibship inference from multilocus genotype data. Mol. Ecol. Resour..

[65] Pritchard JK, Stephens M, Donnelly P (2000). Inference of population structure using multilocus genotype data. Genetics.

[66] Jakobsson M, Rosenberg NA (2007). CLUMPP: a cluster matching and permutation program for dealing with label switching and multimodality in analysis of population structure. Bioinformatics.

[67] Evanno G, Regnaut S, Goudet J (2005). Detecting the number of clusters of individuals using the software STRUCTURE: a simulation study. Mol. Ecol..

[68] Earl DA, vonHoldt BM (2012). STRUCTURE HARVESTER: a website and program for visualizing STRUCTURE output and implementing the Evanno method. Conserv. Genet. Resour..

[69] Guillot G, Mortier F, Estoup A (2005). GENELAND: a computer package for landscape genetics. Mol. Ecol. Notes.

[70] Schwarz G (1978). Estimating the dimension of a model. Ann. Stat..

[71] Jombart T (2008). adegenet: a R package for the multivariate analysis of genetic markers. Bioinformatics.

[72] Anderson EC, Thompson EA (2002). A model-based method for identifying species hybrids using multilocus genetic data. Genetics.

