# Supplementary material for: Assessment of genetic diversity, population structure and wolf-dog hybridisation in the Eastern Romanian Carpathian wolf population
Source: Sci Rep. 2023 Dec 19;13:22574. doi: 10.1038/s41598-023-48741-x (PMC10730609; doi:10.1038/s41598-023-48741-x)
Supplement: Supplementary file 2 — Supplementary Information 2. [file 41598_2023_48741_MOESM2_ESM.pdf]

Supplementary Information

Assessment of genetic diversity, population structure and wolf-dog hybridisation in the Eastern Romanian Carpathian wolf population

Anne Jarausch [1,2,*], Alina von Thaden [1,3], Teodora Sin [4,5], Andrea Corradini [5,6,7], Mihai I. Pop [5], Silviu Chiriac [8], Andrea Gazzola [5], Carsten Nowak [1,3]

[1] Conservation Genetics Group, Senckenberg Research Institute and Natural History Museum Frankfurt, Clamecystraße 12, 63571 Gelnhausen, Germany

[2] Department of Biological Sciences, Johann Wolfgang Goethe-University, Biologicum, Max-von-Laue-Straße 9, 60438 Frankfurt am Main, Germany

[3] LOEWE Centre for Translational Biodiversity Genomics (LOEWE-TBG), Senckenberganlage 25, 60325 Frankfurt am Main, Germany

[4] Department of Systems Ecology and Sustainability, Faculty of Biology, University of Bucharest, Splaiul Independentei 91-95, 050095 Bucharest, Romania

[5] Association for the Conservation of Biological Diversity, Ion Creanga 12, 620083 Focsani, Romania

[6] Current address: Animal Ecology Unit, Research and Innovation Centre, Fondazione Edmund Mach, Via Edmund Mach 1, 38098 San Michele all'Adige, TN, Italy

[7] Current address: NBFC, National Biodiversity Future Center, 90133 Palermo, PA, Italy

[8] Environmental Protection Agency, Vrancea County, Dinicu Golescu 2, 620106 Focsani, Romania

*Correspondence: Anne Jarausch, jarausch.anne@gmail.com

Table of contents

Title	Page
Supplementary Results: Species identification and mtDNA sequencing	2
Fig. S1: Analyses of wolf population structure	3
Fig. S2: PCoA analysis of putative wolves and dogs from Romania using SNPs	4

Supplementary Results

Species identification and mtDNA sequencing

We identified control region (CR) haplotypes for 406 out of 444 (91.4%) mostly non-invasively collected samples in the study areas and the surrounding area. CR sequencing of 32 samples (7.2%) resulted in other species (red fox (*n* = 2), wild boar (*n* = 1), roe deer (*n* = 1), brown bear (*n* = 5), lynx (*n* = 6), horse (*n* = 1), domestic cat (*n* = 3) and domestic dog (*n* = 13)).

Two samples were not mtDNA sequenced, as the haplotype for the two individuals had already been identified by another sample. In four cases where mtDNA sequencing was not successful, the haplotype for the relevant individual (RW001f, RW006f, RW059f and RO028m) had been identified by another sample. For RW053f, mtDNA sequencing was not successful, but the haplotype could be assigned trough pack reconstruction (Mother and pups carried H14).

No haplotype could be identified for one individual. Two samples were identified as wolf samples, but without successful haplotype identification. Species identification was not successful for 38 samples (8.6%).

Supplementary Figures

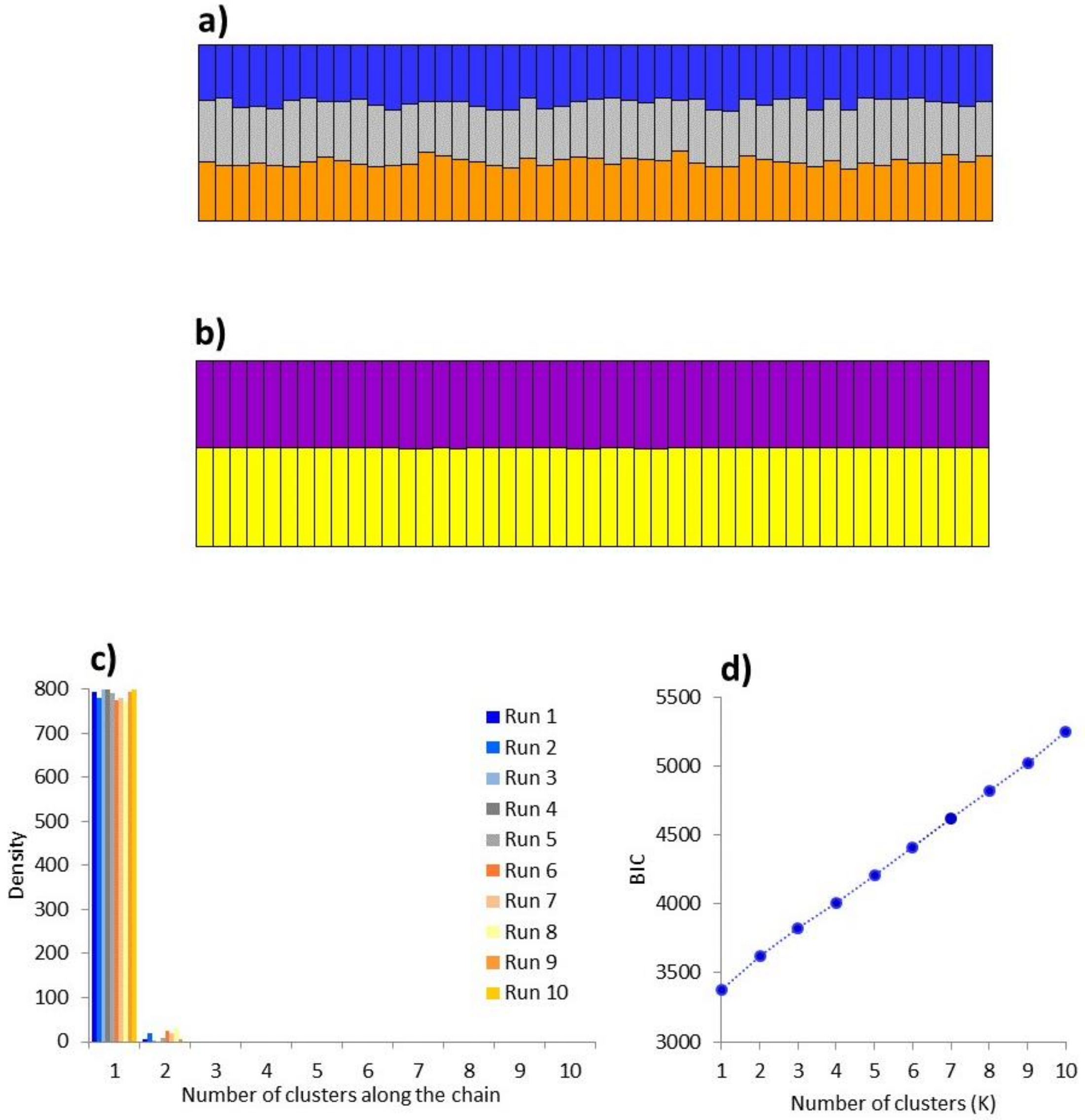

Figure S1. Analyses of wolf population structure within the study region based on 13 microsatellites. a) STRUCTURE results for *K* = 3 and b) GENELAND results for the most supported *K* = 2 based on ten initial runs with a spatial prior and correlated allele frequency model. Each individual is represented by a vertical bar partitioned into coloured segments according to the membership proportions of the inferred clusters. c) Number of clusters inferred by ten initial GENELAND runs with a non-spatial prior and correlated allele frequency model, d) Optimal number of clusters (*K*) based on the Bayesian information criterion (BIC).

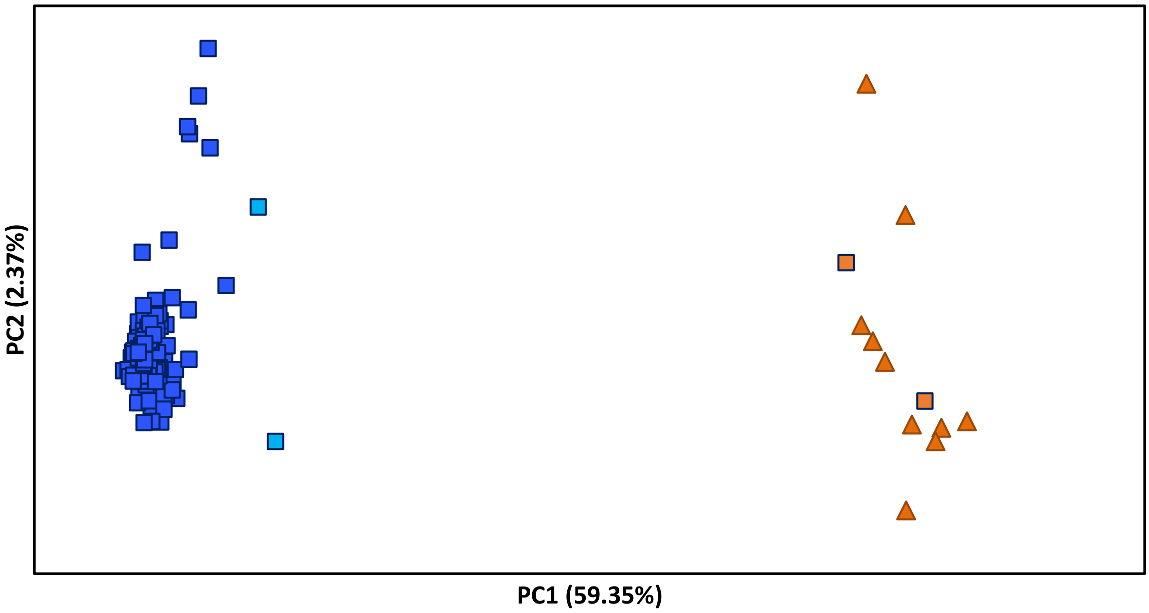

Figure S2. PCoA analysis of putative wolves (*n* = 127) and dogs (*n* = 10) from Romania based on 94 ancestry informative SNPs. Each dot represents an individual genotype of putative wolves (squares) or reference dogs (triangles). Individual dots are coloured according to the highest assignment probabilities based on the NewHybrids analysis (wolves in blue, dogs in orange and the two potential second generation backcross to wolf hybrids [BCW2] in cyan).
